# Precipitation and Primary Health Care Visits for Gastrointestinal Illness in Gothenburg, Sweden

**DOI:** 10.1371/journal.pone.0128487

**Published:** 2015-05-28

**Authors:** Andreas Tornevi, Lars Barregård, Bertil Forsberg

**Affiliations:** 1 Department of Public Health and Clinical Medicine, Division of Occupational and Environmental Medicine, Umeå University, Umeå, Sweden; 2 Department of Public Health and Community Medicine, Division of Occupational and Environmental Medicine, University of Gothenburg, Gothenburg, Sweden; The Australian National University, AUSTRALIA

## Abstract

**Background:**

The river Göta Älv is a source of freshwater for the City of Gothenburg, Sweden, and we recently identified a clear influence of upstream precipitation on concentrations of indicator bacteria in the river water, as well as an association with the daily number of phone calls to the nurse advice line related to acute gastrointestinal illnesses (AGI calls). This study aimed to examine visits to primary health-care centers owing to similar symptoms (AGI visits) in the same area, to explore associations with precipitation, and to compare variability in AGI visits and AGI calls.

**Methods:**

We obtained data covering six years (2007–2012) of daily AGI visits and studied their association with prior precipitation (0–28 days) using a distributed lag nonlinear Poisson regression model, adjusting for seasonal patterns and covariates. In addition, we studied the effects of prolonged wet and dry weather on AGI visits. We analyzed lagged short-term relations between AGI visits and AGI calls, and we studied differences in their seasonal patterns using a binomial regression model.

**Results:**

The study period saw a total of 17,030 AGI visits, and the number of daily visits decreased on days when precipitation occurred. However, prolonged wet weather was associated with an elevated number of AGI visits. Differences in seasonality patterns were observed between AGI visits and AGI calls, as visits were relatively less frequent during winter and relatively more frequent in August, and only weak short-term relations were found.

**Conclusion:**

AGI visits and AGI calls seems to partly reflect different types of AGI illnesses, and the patients’ choice of medical contact (in-person visits versus phone calls) appears to depend on current weather conditions. An association between prolonged wet weather and increased AGI visits supports the hypothesis that the drinking water is related to an increased risk of AGI illnesses.

## Introduction

Precipitation can be a risk factor for acute gastrointestinal illnesses (AGI), as runoff increases the chances that pathogens are entering freshwater supplies. The majority of reported AGI outbreaks in developed nations worldwide were preceded by events of heavy rainfall [1–3]. A few studies have also reported associations with rainfall and AGI during periods when no outbreak was registered [4–7], and it has been argued that the effect of contaminated drinking water on the incidence of AGI is largely unknown [8]. Various studies that aimed to assess associations between AGI and factors related to drinking-water quality have used data from hospital admissions [9–11] and outpatient visits to emergency rooms or physicians [12, 13]. As patients with AGI usually do not need hospital care or prescriptions, such data are likely to largely underestimate the true incidence of AGI. In a previously published paper, we studied the effect of precipitation on the daily incidence of AGI using the number of telephone calls to a medical-advice center (nurse advice calls) [7] within the City of Gothenburg, Sweden, and the study reported increased calls regarding AGI symptoms on days characterized by wet weather and during a lag period of five to six days after heavy rainfall events. This present study aimed to investigate the effect of rainfall on the number of visits to primary health-care centers owing to gastrointestinal illnesses and targeted the same population and covered the same period as our previous study [7]. Another aim was to compare these two different types of contacts and health-data sources (AGI nurse advice calls and AGI visits to health-care centers), evaluating similarities and differences in their seasonal patterns and their short-term associations.

Climate projections for most areas in Sweden predict more days of wet weather and more events of heavy rainfall, and according to the Swedish Climate and Vulnerability Assessment Report, increased precipitation will have a direct impact on drinking-water quality [14] and potentially on public health. Thus, there exists a need for more data concerning the effects of precipitation on AGI as a basis for preventive measures.

## Methods

### Ethical Statement

This research article is part of a research project named: Climate Change, Fresh Water Quality, Treatment and Distribution- Assessment of Microbial Risks from Health Studies. The project has been approved by The Regional Ethical Review Board—Division of Medical Research, Umeå, Sweden (Dnr: 2010-259-32M). The Regional Ethical Review Board waived the requirement for participant consent since this project only uses anonymized register data. This research was conducted in Sweden.

### Study area

The City of Gothenburg (latitude ~ 58, longitude ~12) is Sweden’s second largest city with a population of around ½ million. There are two municipal drinking water utilities in Gothenburg, Alelyckan (AWU) and Lackarebäck (LWU), and under normal conditions they use different freshwater sources, the river Göta Älv and a lake system (Delsjöarna), respectively. However, to maintain water level in the lake system it is being filled with river water, pumped via a 9 km long tunnel, with a travel time about two days [15]. Upstream AWU the river is at risk of being exposed to rainfall-runoff from agricultural land, and contamination from overflowing sewer systems in events of heavy rainfall. Raw water quality parameters is repeatedly measured at several locations along the river, and when river water is considered unsuitable for drinking-water production, for example during high turbidity or when high levels of indicator bacteria is detected, the river water intake at AWU can be closed, and AWU is instead supplied with water brought back through the tunnel. Thus, longer closures mean that both AWU and LWU are supplied with water from the lake system.

AWU distributes drinking water to households in the northern area of the city and the southern part of the city obtains drinking water from LWU. As the drinking-water distribution net is interconnected in the city, the central parts are supplied with a mixture of water from the two utilities. The drinking water utilities use a conventional water treatment technique with barriers consisting of: chemical flocculation with alum and sedimentation, rapid filtration through granular activated carbon, and disinfection with chlorine/chlorine-dioxide.

With respect to the northern latitude of the study area, the climate is mild. February is usually the coldest month with daily mean temperatures a few degrees Celsius below zero. The river is usually not covered with ice during winter seasons, and precipitation is fairly constant throughout the seasons although the second half of the year generally experiences more and heavier rainfall events.

### Data

Data on all visits to primary-care centers within the City of Gothenburg that took place between 1 January 2007 and 31 December 2012, with ICD codes related to gastrointestinal illnesses (ICD: A00–A09), were obtained from the county health-care administration (these data are henceforth referred to as *AGI visits*). The ICD codes A00–A02 and A05 (S1 Table) were relatively few in number and were considered very unlikely to be drinking-water related; therefore, they were excluded. For each patient’s visit, the data included an area code which was used to match visits to the AWU distribution area. The municipal water organization in Gothenburg (Kretslopp och Vatten, Göteborg stad) used hydrological and geographical models to define the area, resulting in an accuracy of about 75%, meaning that 75% of the time, the area received drinking water from AWU alone. We refer to this division of the city in terms of an *AWU area* and an *LWU area*; the latter includes a larger part of the population living in the mixed-water zone.

Data on nurse advice calls related to AGI (vomiting or nausea, stomach pain, diarrhea) were obtained from individuals registered at households within the City of Gothenburg between 28 November 2007 and 31 December 2011 and were matched to the AWU area with a geographical precision of 95% (house level), as reported previously [7].

Daily precipitation observations from a meteorological station about 30 kilometers upstream Alelyckan (Alvhem) were obtained from the Swedish Meteorological and Hydrological Institute. Prior analyses have shown that this station is preferable for predicting levels of indicator bacteria outside the river-water intake [16].

### Statistical analysis

Analyses of short-term effects on the outcome were conducted with time-series regression using generalized additive models (GAM), adjusting smoothly for seasonal and long-term trends and controlling for day of the week and national-holiday effects. We stratified the analyses in cases defined as AWU with the aim of targeting the population receiving AWU drinking water, and for comparison, analyses were also conducted on the population in the LWU area and for the total population in the City of Gothenburg (i.e., including all visits).

Daily counts of the outcome were assumed to follow a Poisson distribution, and a general expression of the model could be written as:

$$\begin{array}{l} Number\;of\;daily\;events∼Poisson(\lambda_{t})\\ \text{log}(\lambda_{t})=\alpha +s(v_{t}|df)+\gamma DOW_{t}+\gamma_{1}HD_{t-1}+\gamma_{2}HD_{t}+\gamma_{3}HD_{t+1}+f(x_{t(L)})\\ \;=\alpha +COVs+f(x_{t(L)}) \end{array}$$ (1)

where the subscript *t* corresponds to each day in the data. The function *s* represents a thin-plate spline and adjusts for seasonal patterns and time trends with the variable *v*, representing an ordered discrete count of the days in the data. The flexibility parameter in *s* was set to about seven degrees of freedom (df) per year. Day of the week (*DOW*) and national holidays (*HD*) represent indicator variables. These variables are regarded as covariates (*COVs*), while the function *f* represents an association of a predictor (*x*) of particular interest: effects of daily precipitation over a lag period, the effects of consecutive days of dry or wet weather, or an association with nurse advice calls. Thus, the function *f* was set up differently depending on the exposure variable.

#### Precipitation’s effect on AGI visits

To examine if prior precipitation is associated with the number of AGI visits, we used a statistical method similar to that applied in our study of AGI calls [7]. Daily precipitation was modeled with a distributed lag nonlinear predictor (DLNM) [17] on AGI visits. In DLNM models, *f*(*x*_*t*(*L*)_) in (1) represents a two-dimensional function spanning the lag space (L days prior to *t*) and in space of precipitation (*w*). Length of lag period has to be predefined, and since different pathogens have different incubation periods, and despite our previous paper [7] reported only associations within the first week after rainfall it cannot be assumed that these two types of medical contacts represent similar lag structure, symptoms, age groups, or severity of illnesses, we evaluated lag periods of 0–15, 0–21, and 0–28 days.

We used natural cubic splines describing associations along lags and with amount of rainfall (non-linear associations). We also evaluated whether a linear or categorical relationship in exposure space was more adequate. Thus, we tested different numbers of spline-knots in the lag space, allowing for detecting associations along whole lag space, with different approaches in the exposure space, and assessed a design that resulted in a minimum AIC score.

Additionally, we considered whether consecutive days of wet or dry weather affected daily AGI visits by considering a wet day as *w* > 0 and creating a variable (*u*_*t*_) for consecutive weather conditions. This exposure variable was considered as a factorial variable, and to keep the number of factor levels lower we separated consecutive weather conditions into six categories, and choose the marginal levels to hold about the same number of observations. These two types of precipitation predictors (DLNM and consecutive weather) were analyzed in separate models, but we also investigated potential effect modifications (possibly owing to collinearity) by including them simultaneously in the same model. Furthermore, we studied the influence of potential confounding effects resulting from long-term and seasonal variations by relaxing our seasonal component between 3 and 12 df/year.

#### Association between AGI calls and AGI visits

To assess short-term relations between the two types of AGI health data, we examined whether the daily AGI calls could be considered a predictor of AGI visits within the seven following days. The analyses were performed in a two-stage approach: AGI calls were first adjusted for long-term variation, day of week, and holiday effects (*COVs* in (1)). In the second stage, the unscaled residuals (*r*) from the first stage were evaluated as independent predictors for each day in the preceding week (an unconstrained distributed lag design), with the AGI visits as outcome, once again using (1). Thus, when analyzing a linear relation between AGI calls and AGI visits, function *f* in (1) becomes $f(x_{t(L)})=\sum_{i=0}^{7}\beta_{i}\times r_{t-i}$, where the coefficient *β*_*i*_ represents the effect on the outcome at lag *i*.

As a sensitivity analysis, we checked the robustness of the estimates by varying the flexibility in the seasonal component between 3 and 12 df/year in both stages independently, and we evaluated a possible nonlinear relationship by using a penalized thin-plate spline restricted to four degrees of freedom ($f(x_{t(L)})=\sum_{i=0}^{7}s(r_{t-i}|df_{i}\leq 4)$).

The two AGI data sets were also compared with respect to long-term variations. The number of daily AGI visits to clinics and AGI calls were then considered as total counts from a binomial distribution, and we fit a GAM model with AGI visits as ‘successes’ and evaluated how the ratio varied within season and with year by including factors for *month* and *year*. The model was also adjusted for *DOW*, and due to findings from the DLNM models we included daily precipitation (lag 0). We excluded weekends and national holidays from the data to only analyze days with normal opening hours at clinics.

All statistical analyses were carried out in R 2.15.1 [18], using MGCV [19] and DLNM [17] packages.

## Results

### Descriptive and long-term variations

The total count of visits to primary health-care centers that resulted in diagnoses of ICD A03–04 or A06–A09 in Gothenburg between 1 January 2007 and 31 December 2012 was 17,030 cases. A09 (*Infectious gastroenteritis and colitis*, *unspecified*) was the dominant ICD code (95%). AGI visits related to the AWU area accounted for 43% of the total count. Daily precipitation in the study period ranged 0–54 mm, with an average of 3.3 mm per day, or 7.2 mm when excluding dry days. Descriptive statistics of AGI visits and precipitation are displayed in Table 1. S1 Table displays monthly statistics of AGI visits, and S2 Table total counts per ICD code.

**Table 1:** Descriptive statistics AGI visits and weather.

	Min	5th	10th	25th	50th	75th	90th	95th	Max	Mean	SD
AGI visits (ICD A00-A09)											
City of Gothenburg	0	0	0	3	7	12	17	20	37	7.9	6.3
AWU population	0	0	0	1	3	5	7	9	16	3.4	2.9
LWU population	0	0	0	1	4	7	10	12	26	4.5	4.1
Weather											
Precipitation (mm)	0	0	0	0	0	4.2	10.8	16.2	54.3	3.3	6.2
Precipitation (mm), dry days excluded (54%)	0.1	0.3	0.6	1.9	4.8	10.1	16.9	23.2	54.3	7.2	7.5
Consecutive wet days (dry days excluded)	1	1	1	1	2	4	6	8	14	2.8	2.3
Consecutive dry days (wet days excluded)	1	1	1	1	3	5	10	13	38	4.3	4.9

Descriptive statistics, (percentiles, mean and standard deviation (SD)), of daily visits to primary health care centers diagnosed ICD A00-A09, and precipitation observations, in the period 2007–2012.

AGI visits showed an increasing trend until 2010. On average, there were about 8.5 times more AGI calls than AGI visits during the period 2008–2009, and there were about 5 times more AGI calls than AGI visits in 2010–2011.

Seasonal differences between AGI visits and AGI calls were concluded. The daily number of calls peaked in winter periods (February–March) with at close to twice the number recorded for June–September. AGI visits also showed an increase during winter periods but had the highest monthly average in August, which saw 1.5 times more visits than June did. Thereby, the smallest difference in frequency between the two types of AGI data occurred in August, and the largest difference occurred in February. The binomial GAM model estimated a statistical difference in seasonal patterns in AGI visits relative AGI calls. Using February as reference month, an odds ratio (OR) for August was estimated to 2.2 for AGI visits relative AGI calls (95% confidence interval (CI): 2.0–2.4).

**Fig 1:**
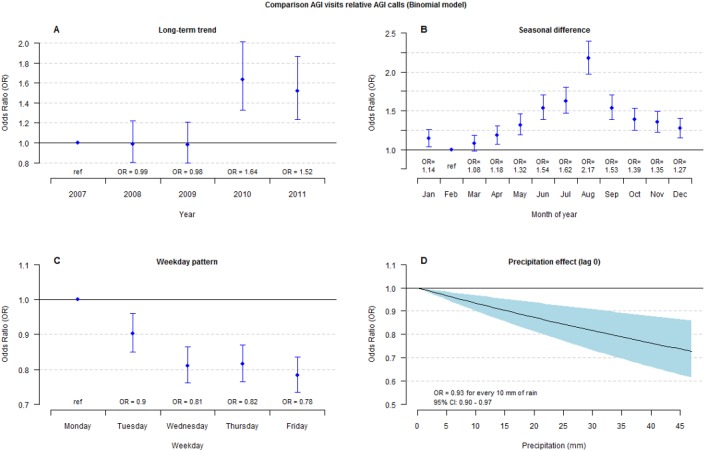
AGI visits relative AGI calls. Comparison of AGI visits relative AGI calls using a binomial regression model including all data (City of Gothenburg). A. Long term trend—estimated odds ratios by year. B. Seasonal difference—estimated odds ratios for month of year. C. Week day effect—estimated odds ratios for weekdays. D. Estimated odds ratio of precipitation lag 0. Bars and filled area represent 95% confidence intervals.

During weekends, the number of AGI visits was very low (mainly due to limited opening hours), while AGI calls reached their lowest numbers on Fridays. Both AGI visits and calls had the highest weekday mean value on Mondays, and the increase was relative higher in visits. Daily observations, trends, and monthly ratios between AGI visits and AGI calls are illustrated in S1 Fig, and odds ratios for the relative difference between AGI visits and AGI calls by year, month of year and weekdays, are displayed in Fig 1.

### AGI visits and precipitation

**Fig 2:**
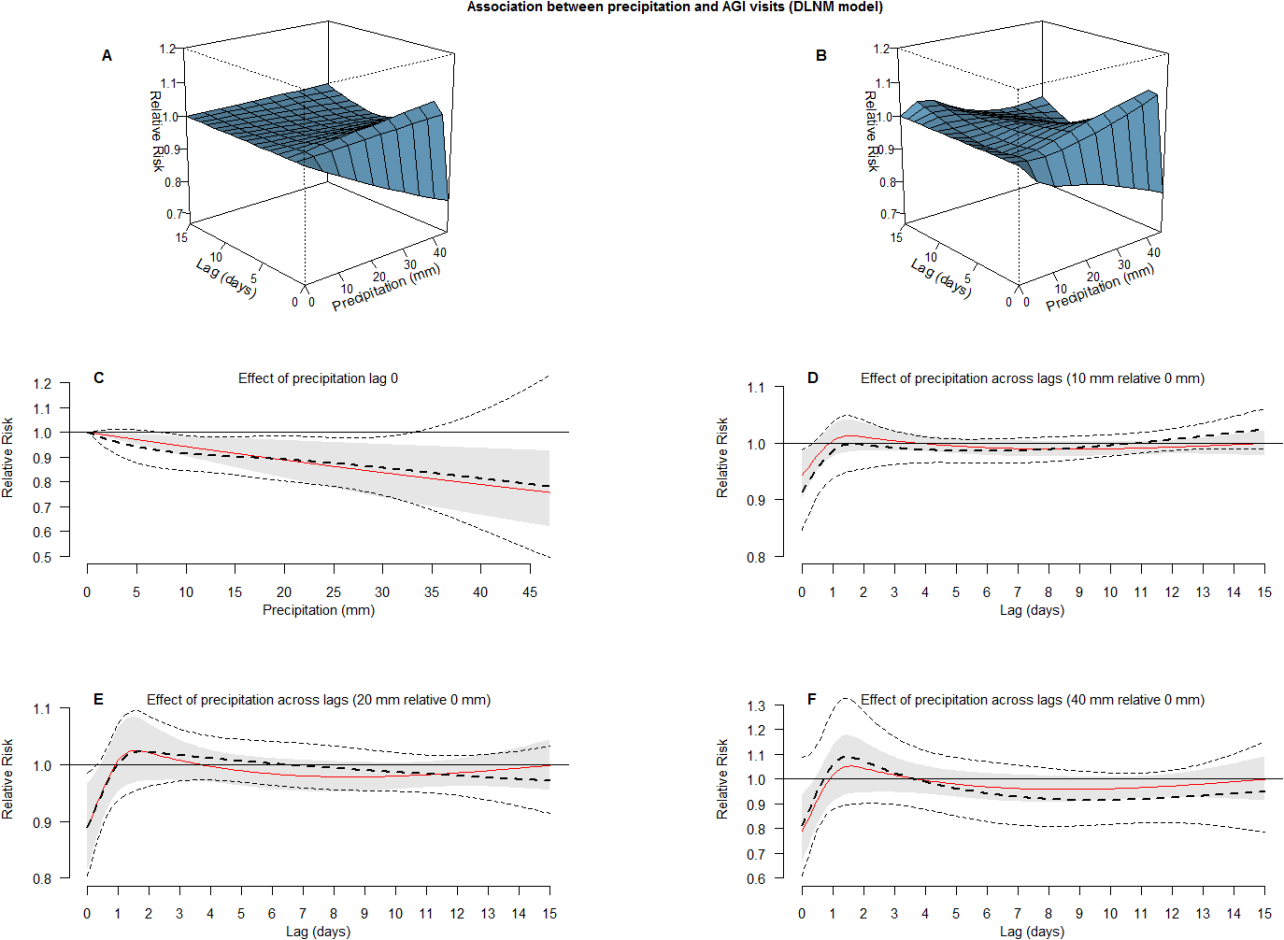
Association between precipitation and AGI visits. Precipitation effects on daily number of AGI visits (AWU area) produced from two DLNM models, linear (shaded) and non-linear (dotted lines) in space of precipitation. A. 3-D surface illustrating linear effects of precipitation along lags. B. 3-D surface illustrating non-linear effects of precipitation along lags. C. Effect of precipitation at lag 0 with 95% confidence intervals. D. Effect of 10 mm precipitation along 0–15 lags with 95% confidence intervals. E. Effect of 20 mm precipitation along 0–15 lags with 95% confidence intervals. F. Effect of 40 mm precipitation along 0–15 lags with 95% confidence intervals.

The number of daily AGI visits was in the DLNM model associated with a decrease was on the day of the precipitation event (lag 0). The association was best fitted (according to AIC score) with a linear relationship; for every 10 mm a relative risk (RR) of 0.95 (95% CI: 0.91–0.98) were estimated for the AWU area. Analyzing LWU area the RR was 0.96 (95% CI: 0.92–0.99), and 0.95 (95% CI: 0.93–0.98) when including all visits. Fig 2 illustrates results from a DLNM models allowing for linear, and nonlinear, associations in lag space and in space of precipitation, and no significant delayed effects of precipitation events were found.

**Fig 3:**
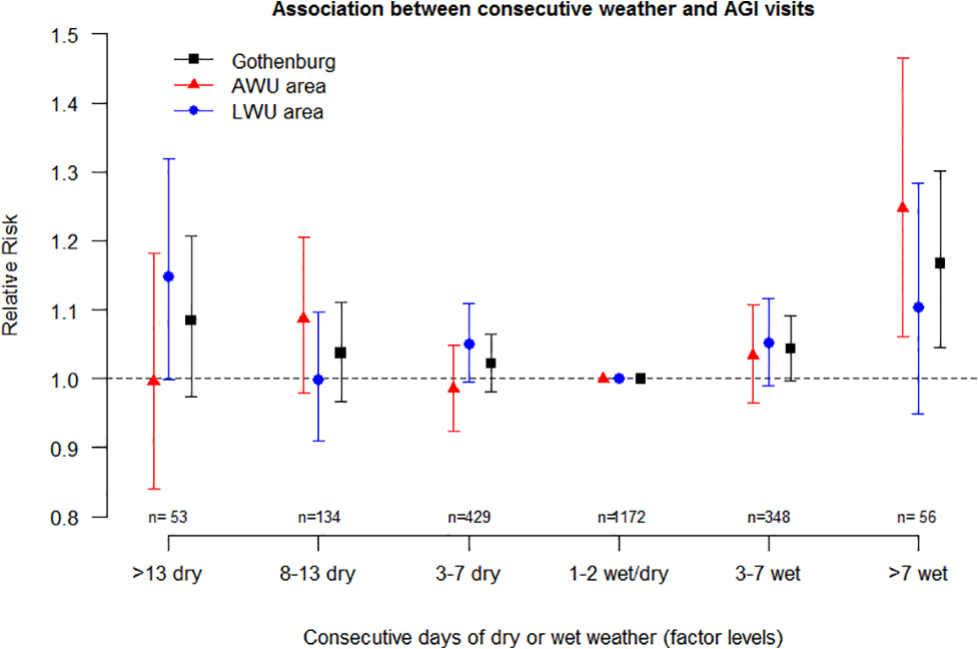
Association between consecutive weather and AGI visits. Effect of consecutive days of wet or dry weather on AGI visits. Bars represent 95% CIs, and *n* is the number of observation days in each factor level. One or two consecutive wet or dry days are set as reference level.

Consecutive days of precipitation were observed to have an effect on the daily number of AGI visits. When the analysis was restricted to the AWU population, a significant increase of 25% (RR = 1.25, 95% CI: 1.06–1.47) was estimated for more than seven wet days in relation to the reference (one or two days of consecutive dry or wet weather). The same statistic was +17% (RR = 1.17, 95% CI: 1.05–1.30) analyzing all visits but was non-significant among the LWU population (RR = 1.10, 95% CI: 0.95–1.28). Fig 3 illustrates the estimates of consecutive dry and wet weather for all factor levels and areas.

### Short-term association between AGI calls and AGI visits

**Fig 4:**
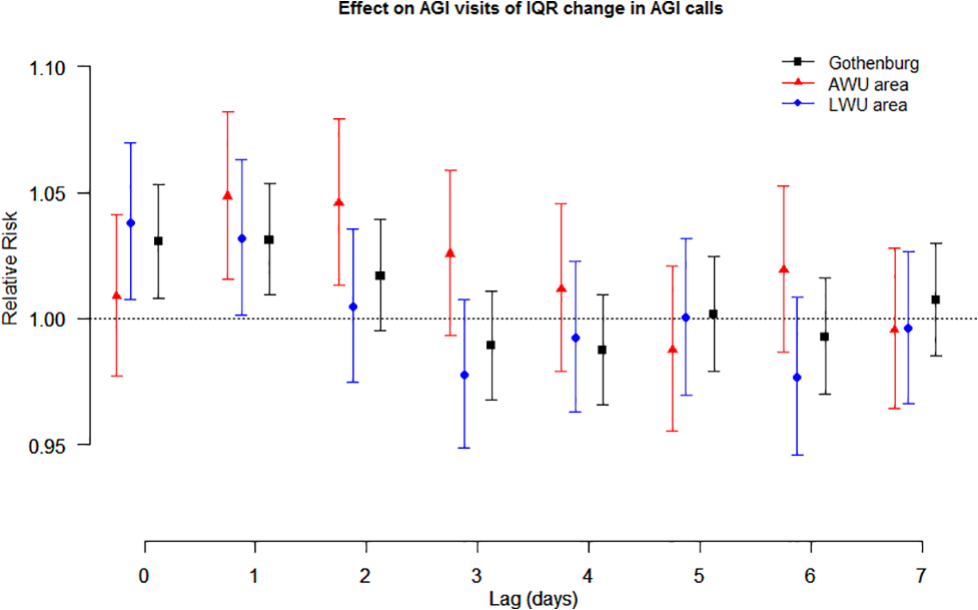
Short-term effect AGI calls on AGI visits. Relative risks of AGI visits across 0–7 lag days of an IQR increase of AGI calls. Bars represent 95% confidence intervals.

A limited short-term association was found between the number of AGI calls and AGI visits. An interquartile range (IQR) increase in the daily number of AGI calls (~10 calls/day) corresponded to an increase of 3% (95% CI: 1%–5%) in AGI visits at lag 0 and lag 1. Restricting the analyses to the AWU area, an IQR increase in AGI calls (about five calls per day) was associated with an increase of about 5% in AGI visits at lag 1 (95% CI: 2%–8%) and lag 2 (95% CI: 1%–8%). The short-term associations at lag days 0–7 for all areas are illustrated in Fig 4.

### Sensitivity analysis

A DLNM model was best designed (according to AIC scores) with 2 knots early in lag space (within lag 3) and with a linear relationship with precipitation. When the consecutive-weather predictor and the DLNM predictor were included in the same model, the DLNM predictor showed associations similar to those found when included alone, while the effect estimate of consecutive wet weather (more than seven wet days) increased 2–3 percentage points. Varying the long-term trend adjustment from 3 to 12 df/year altered the estimate of the increase after wet periods (more than seven wet days) to between 12% and 19%, studying all visits. A similar sensitivity analysis of short-term associations between AGI calls and AGI visits showed only limited modifications.

## Discussion

Our comparison of AGI calls and AGI visits displays only weak relationships. AGI calls exhibit a clear seasonal pattern, peaking in winter periods, which were not equally distinct in AGI visits. The increased incidence of AGI in winter periods is likely a reflection of the seasonality of viral (especially Norovirus) infections. The recommendation from health authorities is to stay at home so as to limit the spread of infections and to visit health-care centers only if symptoms persist over a long period, if signs of dehydration appear (especially in small children), or if the patient is vulnerable because of other medical conditions. In addition, our AGI visits data may not include the most vulnerable patients, who may instead visit hospital emergency departments. In a study on the population in Milwaukee, Wisconsin, AGI cases at emergency departments displayed distinct winter peaks [6] probably caused by the seasonality of viral infections. In a large data study from the US on hospitalizations due to gastrointestinal infections, seasonality of different predominant pathogens were analyzed and concluded both viral infections and unspecified infections peaking concurrently in winter month [20]. It is thereby likely that viral infections are over-represented within unspecified infections, and viral infections are considered the major cause of waterborne illnesses worldwide [21].

AGI visits also presented a more concentrated peak in August, a pattern that was not observed in AGI calls. The reason for the increase in August is unclear, but one might speculate that some cases arise when people returning from summer vacations abroad seek medical treatment for AGI symptoms, or because medical contacts are postponed in July, the peak vacation period in Sweden. August is also a month with optimal conditions for bacterial growth in food owing to high temperatures. This explanation, however, is not supported by the lack of such an increase in AGI calls in August. Overall, these dissimilarities in seasonal patterns between the two types of AGI-incidence data suggest that they partly represent different types of gastrointestinal illnesses.

Results of the short-term association analysis show an increase in AGI visits on the same day or a few days after elevated AGI calls. These associations were weak, but estimates are likely affected by the opposite effects found for the first days of precipitation, when AGI visits decreased but AGI calls increased [7]. This relation was also found in the binomial model where precipitation influenced the proportion of visits relative calls negatively (Fig 1 D). Thus, it appears that weather conditions affect the choice of medical contact. On a rainy day, the choice might shift from visiting a health-care center to making a telephone call for advice. It is reasonable to consider that the visit is thereby just postponed to the next day. The DLNM model indicated an increase at lag 1–2 was but it was not statistically significant.

Short-term associations between AGI visits and AGI advice calls are also affected by differences in availability. The nurse advice line is always open, 24 hours a day, whereas most primary health-care centers are closed during evenings and nights, and many are also closed on weekends. Patients needing medical care at these times are sometimes directed to emergency rooms or, on weekends, are advised to wait until Monday.

The increasing trend in AGI visits in the middle of our study period is likely to be in part the result of reorganization of health-care centers and of changing practices in reporting the statistics to the county administration. Thus, a limitation of this study is the low frequency of daily visits, especially in the first years. This restricts the chances of identifying relationships beyond the normal variability in the data. National telephone interviews (n = 1,000) conducted by the Swedish National Food Agency found that 9% of those surveyed thought they would use the nurse advice line for medical guidance during an AGI event [22]. A follow-up study by the same agency indicates that this number overestimates how often people actually call, but applying this estimate to the City of Gothenburg suggests that less than 2% of those who experience a gastrointestinal illness will visit a primary health-care center seeking treatment for it, since we observed about five times more calls than visits during the most recent years.

The finding associating increased AGI visits with prolonged wet weather aligns with our previous observation when studying AGI calls [7]. This increase could be a result of poor drinking-water quality, as rainfall has been shown to indicate increases of pathogens concentrations in the fresh water supply [16], causing potential risk of treatment failures in the drinking water production. Poor infrastructure in the distribution system could also lead to intrusions of pathogens, especially in events of rainfall. However, the increase of visits with prolonged wet weather might also be explained by other causes. During rainy weather people are likely to spend more time indoors, and more people may use public transportation. Such behavioral patterns in theory increase the transmission risk of communicable diseases. We found, however, the association between wet periods and AGI visits to be more pronounced in the distribution area that received drinking water mainly from river water, which water quality is strongly related to wet or dry weather [16]. Therefore, we think that contaminated water is a more likely explanation than general behavior is. Other studies have related cumulative rainfall to drinking-water-related increased AGI incidence, regarding both outbreaks and normal endemic levels. A study conducted in the United States reported that the majority of reported outbreaks of gastrointestinal illnesses during the period 1948–1994 were preceded by periods of high monthly precipitation [1]. Cumulative rainfall over five days has been positively associated with waterborne disease outbreaks in Canada in the years 1975–2001 [3]. In a large time-series study on laboratory-confirmed cryptosporidiosis in England (1990–1999), the cumulative precipitation over seven days in the northwest region was shown to increase the number of such cases [4]. In a study of 89 drinking-water-related outbreaks (1910–1999) in England and Wales, the cumulative rainfall over seven days was associated with increased risk [2]. The same study also linked low rainfall with increased risk of outbreaks, and several different causal factors were discussed [2]. In this present study an indication of elevated number of AGI visits was seen with dry periods especially for the LWU area (borderline significant). In our case the LWU fresh-water supply is a lake surrounded by recreational areas with bathing places, picnic sites and tracks, why dry weather likely associates with increased human activity in the area, implicating elevated risks that pathogens are introduced into the water. However, the lake is supplied with river water to maintain the water level, which makes it difficult to speculate about the causes behind this possible association between AGI visits and dry weather.

To summarize, this study indicates an increase in AGI visits during prolonged periods of wet weather, but it does not support the claim that an arbitrary event of heavy rainfall would lead to a delayed increased number of AGI visits, as observed in the study of AGI calls [7]. The previously identified lag period of increased AGI calls after heavy rainfall seemed to match incubation periods of viral infections, and the comparison of seasonal patterns for AGI visits and AGI calls suggests that calls reflect the incidence of viral infections better than visits do. The results suggest that a wetter climate, expected to accompany climate change, may lead to increasing incidences of gastrointestinal illnesses, but further studies are warranted for a better understanding of the components of this relation, and investments are needed to mitigate risks. Further, we found that a day of wet weather can change the patient’s choice of medical contact from visiting primary-care centers to seeking advice by telephone. This should be taken into account in research using data sources of this kind.

## Supporting Information

S1 Data AGI incidence data. The file includes dates (DATUM), day of week (DOW), precipitation and the data on the daily number of GI calls and GI visits used in the analyses. GI calls and GI visits are separated by area (AWU, LWU and City of Gothenburg (GBG)) and regarding GI visits both the set ‘ICD A00-A09’ and the subset ‘A03-04 and A06-A09’ are provided, where the latter is named ‘_ICDselected’.
(TXT)

S1 Fig Time series of AGI data. A. Daily observations of AGI visits in the City of Gothenburg and smooth spline (white) used for adjusting for long-term and seasonal variations in GAM models. B. Daily observations of AGI calls in the City of Gothenburg and smooth spline (white) used for adjusting for long-term and seasonal variations in GAM models. C. Monthly ratios between the two different types of incidence data.
(TIFF)

S1 Table AGI visits. Descriptive statistics of daily visits to primary health-care centers diagnosed ICD A03-04 or A06-A09 in the period 2007–2012, where N is the number of observation days, Mean is the daily mean value and SE is the standard error of the Mean.
(DOCX)

S2 Table ICD codes. Number of cases diagnosed with ICD A00-A09 in the period 2007–2012, City of Gothenburg, Sweden.
(DOCX)
