# Supplementary figures and images for: Precipitation and Primary Health Care Visits for Gastrointestinal Illness in Gothenburg, Sweden
Source: PLoS One. 2015 May 28;10(5):e0128487. doi: 10.1371/journal.pone.0128487 (PMC4447281; doi:10.1371/journal.pone.0128487)

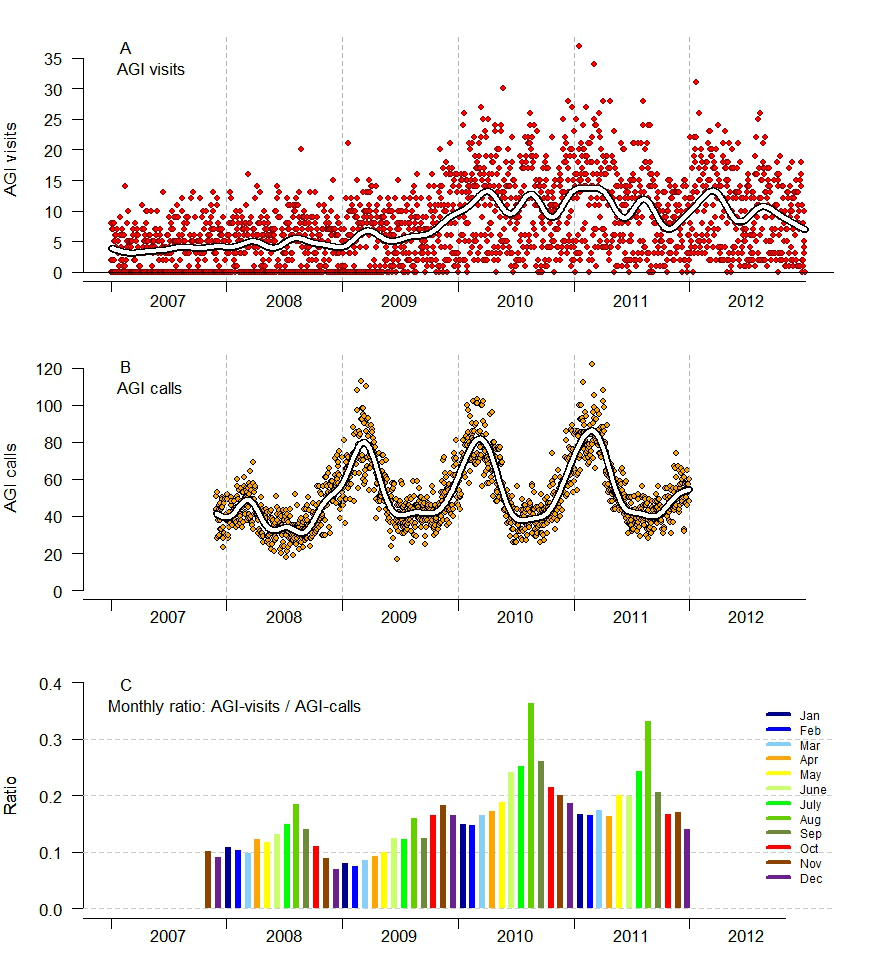

Supplement: S1 Fig — A. Daily observations of AGI visits in the City of Gothenburg and smooth spline (white) used for adjusting for long-term and seasonal variations in GAM models. B. Daily observations of AGI calls in the City of Gothenburg and smooth spline (white) used for adjusting for long-term and seasonal variations in GAM models. C. Monthly ratios between the two different types of incidence data. (TIFF) [file pone.0128487.s002.tiff]
