# Supplementary material for: Precipitation and Primary Health Care Visits for Gastrointestinal Illness in Gothenburg, Sweden
Source: PLoS One. 2015 May 28;10(5):e0128487. doi: 10.1371/journal.pone.0128487 (PMC4447281; doi:10.1371/journal.pone.0128487)
Supplement: S1 Table — Descriptive statistics of daily visits to primary health-care centers diagnosed ICD A03-04 or A06-A09 in the period 2007–2012, where N is the number of observation days, Mean is the daily mean value and SE is the standard error of the Mean. (DOCX) [file pone.0128487.s003.docx]

| *Population* | *Period* | *N* | *Mean* | *SE* | *Min* | *Median* | *Max* |
| --- | --- | --- | --- | --- | --- | --- | --- |
| City of  Gothenburg | 2007-2012 | 2192 | 7.77 | 0.13 | 0 | 7 | 37 |
|  | Jan | 186 | 7.9 | 0.53 | 0 | 6 | 37 |
|  | Feb | 170 | 8.5 | 0.50 | 0 | 7 | 27 |
|  | Mar | 186 | 9.3 | 0.55 | 0 | 8 | 34 |
|  | Apr | 180 | 8.2 | 0.50 | 0 | 7 | 28 |
|  | May | 186 | 7.5 | 0.44 | 0 | 6 | 30 |
|  | Jun | 180 | 6.5 | 0.40 | 0 | 5 | 22 |
|  | Jul | 186 | 7.1 | 0.39 | 0 | 6 | 22 |
|  | Aug | 186 | 9.6 | 0.53 | 0 | 9 | 28 |
|  | Sep | 180 | 6.8 | 0.38 | 0 | 6 | 23 |
|  | Oct | 186 | 6.8 | 0.36 | 0 | 6 | 22 |
|  | Nov | 180 | 7.6 | 0.39 | 0 | 7 | 25 |
|  | Dec | 186 | 7.4 | 0.45 | 0 | 6 | 28 |
| AWU area | 2007-2012 | 2192 | 3.33 | 0.06 | 0 | 3 | 16 |
|  | Jan | 186 | 3.3 | 0.23 | 0 | 2 | 15 |
|  | Feb | 170 | 3.5 | 0.23 | 0 | 3 | 13 |
|  | Mar | 186 | 4.0 | 0.26 | 0 | 3 | 15 |
|  | Apr | 180 | 3.6 | 0.22 | 0 | 3 | 13 |
|  | May | 186 | 3.3 | 0.21 | 0 | 3 | 16 |
|  | Jun | 180 | 2.6 | 0.19 | 0 | 2 | 10 |
|  | Jul | 186 | 3.1 | 0.19 | 0 | 3 | 10 |
|  | Aug | 186 | 4.4 | 0.25 | 0 | 4 | 16 |
|  | Sep | 180 | 3.1 | 0.19 | 0 | 3 | 9 |
|  | Oct | 186 | 2.9 | 0.18 | 0 | 2 | 12 |
|  | Nov | 180 | 3.0 | 0.18 | 0 | 3 | 14 |
|  | Dec | 186 | 3.2 | 0.21 | 0 | 3 | 15 |
| LWU area | 2007-2012 | 2192 | 4.44 | 0.09 | 0 | 4 | 26 |
|  | Jan | 186 | 4.7 | 0.34 | 0 | 3 | 26 |
|  | Feb | 170 | 5.0 | 0.34 | 0 | 4 | 17 |
|  | Mar | 186 | 5.3 | 0.34 | 0 | 4 | 20 |
|  | Apr | 180 | 4.6 | 0.33 | 0 | 3 | 21 |
|  | May | 186 | 4.2 | 0.29 | 0 | 3 | 21 |
|  | Jun | 180 | 3.9 | 0.27 | 0 | 3 | 15 |
|  | Jul | 186 | 4.0 | 0.25 | 0 | 3 | 13 |
|  | Aug | 186 | 5.2 | 0.33 | 0 | 4 | 19 |
|  | Sep | 180 | 3.7 | 0.24 | 0 | 3 | 15 |
|  | Oct | 186 | 4.0 | 0.24 | 0 | 4 | 14 |
|  | Nov | 180 | 4.5 | 0.27 | 0 | 4 | 13 |
|  | Dec | 186 | 4.2 | 0.30 | 0 | 3 | 17 |

**S1 Table. AGI visits.** Descriptive statistics of daily visits to primary health-care centers diagnosed ICD A03-04 or A06-A09 in the period 2007-2012, where *N* is the number of observation days, *Mean* is the daily mean value and *SE* is the standard error of the *Mean*.
