# Supplementary material for: Precipitation and Primary Health Care Visits for Gastrointestinal Illness in Gothenburg, Sweden
Source: PLoS One. 2015 May 28;10(5):e0128487. doi: 10.1371/journal.pone.0128487 (PMC4447281; doi:10.1371/journal.pone.0128487)
Supplement: S2 Table — Number of cases diagnosed with ICD A00-A09 in the period 2007–2012, City of Gothenburg, Sweden. (DOCX) [file pone.0128487.s004.docx]

| ICD | Diagnosis | AWU population | LWU population | Gothenburg |
| --- | --- | --- | --- | --- |
| A00 | Cholera^*^ | 3 | 5 | 8 |
| A01 | Typhoid and paratyphoid fevers^*^ | 0 | 1 | 1 |
| A02 | Other salmonella infections^*^ | 55 | 75 | 130 |
| A03 | Shigellosis^*^ | 2 | 6 | 8 |
| A04 | Other bacterial intestinal infections | 92 | 220 | 312 |
| A05 | Other bacterial foodborne intoxications, not elsewhere classified^*^ | 5 | 23 | 28 |
| A06 | Amebiasis | 42 | 54 | 96 |
| A07 | Other protozoal intestinal diseases | 34 | 37 | 71 |
| A08 | Viral and other specified intestinal infections | 44 | 168 | 212 |
| A09 | Infectious gastroenteritis and colitis, unspecified | 7123 | 9284 | 16407 |
| A00-A09 | Total | 7400 | 9873 | 17273 |

**S2 Table. ICD codes.** Number of cases diagnosed with ICD A00-A09 in the period 2007-2012, City of Gothenburg, Sweden.

^*^Excluded when analyzing precipitation effects (DLNM models).
